# Qualitative study on doctors’ perceived barriers and facilitators towards the practice of delayed prescription: results from focus group discussions in Switzerland

**DOI:** 10.1136/bmjopen-2024-094296

**Published:** 2025-11-04

**Authors:** Aline Rinaldi, Serena Petrocchi, Anna Bullo, Luca Gabutti, Peter Schulz

**Affiliations:** 1 Università della Svizzera italiana, Lugano, Switzerland; 2 Communication, Culture and Society, Università della Svizzera italiana, Lugano, Switzerland; 3 LKC School of Medicine, Wee Kim Wee School of Communication and Information, Singapore

**Keywords:** Antibiotics, GENERAL MEDICINE (see Internal Medicine), QUALITATIVE RESEARCH, Prescriptions

## Abstract

**Abstract:**

**Objectives:**

Antimicrobial resistance is one of the biggest threats posed to healthcare systems, accounting for hundreds of thousands of deaths worldwide and correlated with poorer health status and increased healthcare costs. The practice of delayed prescription seems to be an effective solution to diminish the unnecessary overprescription of antibiotics, as study results demonstrated that it does not negatively affect health status while engaging patients and addressing their desire for a prescription when visiting the doctor’s office. This study investigates the point of view of family doctors practising in Switzerland, a country where delayed prescription has not yet been introduced. The main scope was to describe the perceived barriers and facilitators towards delayed prescription of antibiotics.

**Design:**

A total of five online focus group discussions.

**Setting:**

Family medicine.

**Participants:**

21 family doctors practising in the Italian-speaking region of Switzerland (M=51.24; SD=9.73 years of age; 62% males).

**Results:**

Focus group discussions revealed a generally negative attitude towards delayed antibiotic prescription among participants. Thematic analysis identified three key themes reflecting perceived barriers to its implementation: (1) Maintenance of authority through a gatekeeping role, highlighting concerns about preserving professional control over treatment decisions; (2) Importance of maintaining communication, addressing fears that delayed prescription could undermine clarity and trust in doctor–patient interactions and (3) Healthcare system and guidelines for good practice, which encompasses structural and normative expectations around follow-up visits, pre-existing practices and clinical routines. These themes illustrate the multifaceted nature of physicians’ resistance to adopting delayed prescription in their daily practice.

An additional information that emerged from the discussions is the extensive use in the region of a practice similar to delayed prescription, called ‘the stock antibiotic’. However, it is perceived very differently by physicians because it does not enforce a predetermined waiting time on patients.

**Conclusions:**

Past research has demonstrated that delayed prescription is an effective practice for reducing antibiotic consumption and promoting patients’ empowerment while maintaining their satisfaction. Nevertheless, the results of this study show that doctors’ perceptions of this practice are not always positive. Any attempt to introduce the practice should start with a careful evaluation of the cultural context and doctors’ opinions, as their willingness to embrace the practice is crucial for its successful adoption. A more practical implication of our results stems from the discovery of the practice of the stock antibiotic, which could be described as a new version of delayed prescription, tailored to the customs and practices of the region. This aspect highlights the importance of exploring local contexts to ensure that prescribing practices can be implemented in alignment with local preferences.

**STRENGTHS AND LIMITATIONS OF THIS STUDY**

- The study was conducted with family doctors.
- The track for the focus groups has been developed based on the health belief model theory.
- Results revealed resistance to delayed antibiotic prescription, driven by concerns about professional authority, communication challenges and structural norms within the healthcare system.
- Successful implementation requires considering the cultural context and gaining physicians’ support.

## Introduction

 Antimicrobial resistance (AMR) due to antibiotic use and misuse is one of the major problems that healthcare services and personnel are currently facing, also because of its correlation with poor health outcomes and increasing healthcare costs.^1 2^ AMR already places a significant burden on healthcare systems worldwide, prolonging hospital stays and leading to less effective antibiotic treatments.^2^ As Huemer *et al*^1^ stated in a recent article, antibiotic-resistant bacteria are responsible for 700 000 deaths per year worldwide and will increase significantly by 2050, when it is estimated that around 10 million deaths will be attributable to AMR. Although a precise quantification of its impact remains challenging, AMR represents a growing public health concern in Switzerland as well. According to national surveillance programmes, Switzerland occupies an intermediate position in the European ranking with regard to the prevalence of resistant bacterial strains, reporting lower rates than countries such as France, Italy and the UK, but higher than those observed in Scandinavian countries and the Netherlands.^3^ In response, Switzerland has adopted a comprehensive national strategy aimed at monitoring antibiotic use and preserving their long-term efficacy. This strategy encompasses eight key areas of intervention, the second of which is prevention.^3 4^ It is precisely within this preventive context that the present study is situated.

Overprescription is recognised by the WHO as the main driver of this ‘silent pandemic’, which affects not only developing or low-income countries, but is increasingly becoming a global concern.^5^ In Switzerland, outpatient antibiotic use is relatively low compared with other European countries. Consumption declined starting in 2014 and decreased further during the COVID-19 pandemic, but has since returned to prepandemic levels, with family physicians accounting for 87% of total outpatient use in 2023.^6 7^ Moreover, Switzerland’s healthcare system reflects the country’s cultural, linguistic and socioeconomic diversity,^8^ with regional variations in antibiotic use higher in the French-speaking and Italian-speaking areas than in the German-speaking regions.^6^,^9^

In primary care, the appropriate use of antibiotics is essential to tackling the growing challenge of AMR; in this context, delayed prescribing emerges as a potentially valuable strategy, though not without its challenges. A delayed antibiotic prescription seems to be a valid alternative in those cases where the doctor is not sure that immediate antibiotic treatment is necessary.^10^ It is an antibiotic prescription that becomes available after a waiting time indicated by the treating doctor, usually around 3 or 4 days, and that is to be used only if symptoms worsen or fail to improve by that pre-established waiting time.^11 12^

A delayed prescription has been shown to be effective for most patients presenting with respiratory tract infections (RTIs)^12 13^: the practice does not lead to an increase in symptom duration or severity of illness and generally also results in high rates of patient’s satisfaction.^10 13^ Some studies also demonstrate that, when given the opportunity, patients prefer to adopt a wait-and-see strategy, significantly preferring delayed prescription of antibiotics over immediate prescription.^14 15^

This approach is often used in the context of RTIs, as patients affected by these symptomatologies are recognised as the most demanding in regard to antibiotic treatment.^16 17^ As a result, doctors often prescribe antibiotics in response to perceived patient’ expectations, even though the majority of RTIs are usually self-limiting viral infections.^1018^,^21^

Moreover, studies on the effect of delayed prescription on the rate of antibiotic consumption reported a significant reduction in uptake,^1011 22^,^25^ demonstrating that this method could be a weapon against antibiotic overconsumption and the constant rise of AMR.

One of the characterising aspects of delayed antibiotic prescription is that it requires good communication skills in the context of patient–provider communication and is also a strategy that takes into account psychological and social factors related to patients expecting an antibiotic prescription. Because of these aspects, delayed prescription can be accounted among the methods used in an effective patient-centred care as it complies with the evolution of the practice of the last few decades, with a shift in the patient–provider relationship going towards patients specific needs and promoting shared-decision making interactions.^26 27^

Up until now, existing research has mostly emphasised the positive aspects of this practice and many studies have been performed to explore the effects of its usage on the satisfaction of patients or the reduction in antibiotic consumption.^2328^,^31^ While results pertaining to the use of delayed prescription of antibiotics are promising, there is still a lack of detailed analysis regarding doctors’ attitudes associated with the practice.

In Switzerland, there is no specific prescribing tool that allows for delayed prescriptions, and this practice is currently not in use; moreover, doctors are not notified when patients purchase the prescribed medication. The study area is characterised by doctors primarily using physical prescriptions, handed directly to patients at the end of the visit. When needed, doctors are available to scan and send directly the prescription to pharmacies, so patients do not need to visit the practice solely to collect the prescription. In case of diagnostic uncertainty, the standard practice is that of watchful waiting, which entails the scheduling of a second appointment.

This makes the study area particularly suitable for exploring perceptions of delayed prescription from the perspective of doctors who have not yet implemented or experienced this practice. Examining these perceptions prior to implementation provides a more realistic understanding of initial attitudes that may influence uptake. In contrast, studies conducted postimplementation often capture attitudes shaped by experience, potentially obscuring the challenges and hesitations typically encountered during the early stages of adopting a new practice.

The aim of this study is to identify the perceived barriers and facilitators to the practice of delayed prescription, which will also allow for further generalisation of knowledge on opinions regarding this practice. Results from this study could serve as a resource for informing policy decisions and for assessing the potential for implementing the practice in other countries where it has not yet been adopted.

## Method

### Study design

The qualitative study design was based on focus group discussions with family doctors practising in the Italian-speaking region of Switzerland. Participants in our study were not asked to disclose the ethnicity of their patients; however, the region is home to a diverse population, with a 26% rate of immigrant inhabitants. The most represented nationality is Italian (15%), followed by a few Eastern Europeans and Portuguese. It is also very common for retired citizens from the German-speaking part of Switzerland to move to this region for their retirement.

Through guided discussion, the aim was to explore the physicians’ perceptions of delayed prescription. Since the practice is not used in Switzerland, this setting enabled the study to specifically focus on perceived, rather than experienced, barriers and facilitators, thereby offering insight into practitioners’ unfiltered expectations and predispositions.

Focus groups were chosen as the method for data collection because of the setting, which encourages spontaneous exchange between participants, which could bring up new and innovative topics the researchers could not have been aware of in advance.

This study represents a step undertaken within a larger project that focuses on communication dynamics in the context of family medicine and how they affect antibiotic treatment adherence. The previous step consisted of focus groups performed with the general population^32^ to investigate their attitudes in order to gain a more comprehensive understanding of the two parties involved in the antibiotic prescription process.

### Procedure

Focus groups were performed from 28 September 2023 to 29 November 2023. The selection of participants was performed by a researcher with the help of official cantonal sites providing the names of active and inactive doctors in the area. Contacts extrapolated were later compared with a few people search sites (local.ch; onedoc.ch; …) to exclude those doctors who went into retirement or were not operating anymore in the region. As a first contact with participants, general practitioners (GPs) were then contacted and personally invited to participate in focus groups by phone call and via email. The inclusion criteria for participants were to be operating family doctors, working in a studio situated in the Italian-speaking region of Switzerland, and being able to understand and speak Italian. The focus groups were performed online to accommodate the GPs’ needs and were conducted by two female researchers (SP post-doc researcher; AR, PhD student), one moderator and one assistant, who previously performed focus groups with the general population and have acquired experience in conducting focus groups through their educational background. Participants were asked in advance to return via email a consent form for their participation and a brief questionnaire gathering demographic data.

Each discussion was introduced by the assistant, who explained the main topics and a set of rules to better regulate the online discussion. The researchers introduced themselves briefly by presenting their profile as academics and their interests that led to the present study. The discussion was based on a guide track that was created following the principles of the Health Belief Model,^33^ more specifically the principles of susceptibility, severity, benefits and barriers.

The first set of questions was dedicated to the broad theme of antibiotic use, in particular GPs were asked about the attitudes of their patients towards antibiotics and what they perceived to be the general knowledge on the topic among their patients. Whenever doctors would state a particular attitude, they were asked to describe, if possible, some of the characteristics of their patients sharing said attitude.

The second part was focused more on the practice of delayed prescription. This section was introduced by a brief explanation to make sure that everyone was on the same page, and then GPs were solicited to express their thoughts on the practice through questions about whether they would implement it in their office, and if so, why and in which occasions.

The moderator’s and the assistant’s role during the discussion was to make field notes and solicit any interesting point of view expressed by the participants, to understand whether there was consensus or disagreement, and to make sure that no one was overpowering the other doctors. No other participants were admitted to partake in the discussion.

The discussions lasted for a range going from around 40 min to a maximum of 1 hour and 11 min and there were no repeated discussions. The translated guide used during the focus groups is provided in the online supplemental appendix material A.

Every focus group was held on Microsoft Teams and was recorded with the participants’ consent. One researcher later transcribed the discussions to perform a thematic analysis. Anonymity was granted by substituting the GPs’ names with codes during the transcription phase. In addition, the names of the cities where the offices are located were removed from the transcription to guarantee privacy. The specific codes assigned to participants (P1; P2; P3; …) and focus groups (FG1; FG2; FG3; …) were used to identify participants in the transcriptions and are included at the beginning of each direct participant’s quotation.

Two researchers conducted an inductive thematic analysis independently, which led to the identification of relevant contents in the transcribed text and assignation of a code to each of them to represent significant main themes. The researchers later compared their results and codes and finalised through discussion the names given to the themes. No coding tree was produced, and themes were derived from the data after the conclusion of focus groups. The participants were not contacted again for research purposes, nor were they asked to review the transcripts or comment on the findings.

As approved by the ethical committee, each participating doctor received a CHF 50 voucher.

### Patient and public involvement

Patients or the public were not involved in the design, or conduct, or reporting, or dissemination plans of our research. Doctors were participants of the research project and were recruited as explained in the Method section.

### Sample size

Regarding the sample size, we followed a widely used recommendation for qualitative studies, which suggests continuing gathering data until the point of ‘data saturation’, which means until no new topics emerge from discussion. Following the example of some previous studies,^34^,^38^ it appears that four to six focus groups should be enough to identify the most relevant themes. We also based our estimation on the previously conducted focus groups with the general population,^32^ where it was noticed that by the fourth encounter, the themes were starting to become quite repetitive. As expected, due to workload and lack of time, it was more difficult to recruit family doctors than it had been with the general population in the previous study. We decided on still conducting five focus groups with four to five GPs each. The last two focus groups revealed redundancy in the themes and opinions and confirmed that our estimation to extrapolate main barriers and facilitators was adequate.

## Results

Participants demonstrated a high degree of consistency in their views and attitudes, presenting a largely unified stance toward delayed prescription. This convergence of perspectives was observed across the sample, with no discernible variation associated with age or other demographic characteristics. Overall, the shared perception of delayed prescription was predominantly negative.

All participating physicians expressed opposition to the practice, differing only in the intensity of their stance, thereby presenting a unified front. The absence of variability or polarisation in the opinions expressed allowed for the identification of recurring perceived barriers, which were conceptualised as distinct themes and are reported in detail below. A particularly relevant insight that emerged from discussions concerned a prescribing practice referred to by physicians as the stock antibiotic. Although not formally regulated, this practice appears to constitute an informal local strategy.

The stock antibiotic refers to a practice in which the physician provides the patient with an immediate antibiotic prescription at the time of the consultation, accompanied by verbal instructions to delay its use. Unlike formal delayed prescribing strategies, where mechanisms such as postdated prescriptions can help regulate timing, this approach offers no structural safeguard to ensure that the patient will adhere to the recommended waiting period. Instead, the patient is instructed to begin with symptomatic treatment and to initiate the antibiotic only if symptoms worsen. Physicians typically advise patients to contact them before starting the medication, or, if that is not possible, to notify them promptly after doing so. This strategy relies entirely on the patient’s willingness and ability to follow the oral guidance provided, and therefore presupposes a high level of mutual trust.

Physicians described the stock antibiotic as an exceptional measure, employed in situations of diagnostic uncertainty, or when the patient is unable to schedule a follow-up visit, therefore precluding the application of a wait-and-see approach. They also emphasised that, beyond the clinical context, patients’ cognitive abilities must be carefully considered; this practice is reserved for individuals whom physicians deem particularly reliable and free from any cognitive impairments.

### Participants

21 GPs were involved in a total of 5 focus groups, with age ranging from 35 to 76 years old (mean=51.24; SD=9.73). Most participants were male (62%) and the years they have been practising as family doctors ranged from 2 to a maximum of 40 (M=14.48; SD=11.67). One doctor did not state his postal code, and the others were distributed in the four main regions composing the area of interest for the study, with a prevalence of offices located in the cities. One doctor dropped out right before the start of the discussion because of an unexpected call related event. Participants were familiar with the concept of delayed prescription but had no prior experience using it, as the practice is not currently implemented in the Swiss context.

### Insights on the antibiotic prescription landscape

From the initial phase of our focus group discussions, we were able to gather valuable insights into how family doctors perceive the current landscape of antibiotic prescription in the region. The data suggested that patients’ requests are not perceived as a primary factor influencing antibiotic prescribing behaviour. Several physicians reported a noticeable decline in the frequency of such requests compared with previous years. Moreover, they described a growing level of understanding when the antibiotic is denied.

FG2 / P4: There has been a shift in the general population, a shift of vision or of paradigm, they went from wanting the antibiotic at any small cough to becoming more scientific in their ways to avoid it.

Although the phenomenon of patients requesting antibiotics has diminished, it has not entirely disappeared and was described as a behaviour observed across different patient groups. While some physicians identified certain recurring traits among individuals who are more likely to request antibiotics, there was limited consensus regarding any single defining characteristic. Among the more frequently mentioned attributes were patients originating from Southern Italy or Eastern Europe, with several doctors attributing these patterns to perceived cultural differences in attitudes towards medication and healthcare practices.

FG1 / P2: I also noticed a big discrepancy in expectations in my patients, those coming from the South tend to always want it and those coming from the North are against it.

FG5 / P1: Also Ukrainians lately, even though I can talk to them in their language, it is really hard to get through the message that they don’t need it.

Not only did participants report that patients generally accept the denial of an antibiotic prescription well, but they also expressed a strong awareness of their professional responsibility and authority to withhold such treatments when deemed unnecessary. When we performed our focus groups, several doctors referenced recent professional development events held in the region—one focused on patient adherence and the other on AMR—as influential in shaping their current practices. These experiences appear to have reinforced their commitment to evidence-based guidelines. As a result, participants demonstrated a conscientious approach to prescribing, with many reporting increased caution in antibiotic use, and some noting the adoption of shorter treatment durations tailored to the clinical context and patient condition.

FG2 / P2: […] that’s true, it is not like it used to be in the past when you would give an antibiotic for any small inflammation or infection, the reasoning behind became much more cautious and precise, following also what are the guidelines and norms that are given to us.

FG5 / P1: If there are prerequisites of an infection, at a clinical and laboratory level, I mean from the urine tests, you can prescribe an antibiotic depending on age and co-morbidity of each patient […] I always check antibiotics indications on “First line”, an application developed by infectious disease specialists of our region; they update it depending on the current prevalence of bacteria and their sensitivity to antibiotics in the region.

FG5 / P4: At the moment, if I don’t find the hematology convincing, I don’t prescribe the antibiotic. I have become more cautious about giving antibiotics and the lengths of the antibiotic therapy, as they showed us also on the webinar I attended last week. […] Last week they were talking about a mean duration of five days instead of seven, and I adjusted to this statement.

Following an initial overview of the current perceived state of antibiotic prescribing, the focus group discussions shifted toward a more in-depth exploration of the practice of delayed prescription. Across all groups, participants expressed a predominantly negative attitude towards this approach and articulated well-reasoned arguments to support their stance. Their perceptions were organised into a set of recurring themes that reflected shared concerns and reservations about the appropriateness and effectiveness of delayed prescription in clinical practice.

### Theme 1: maintenance of authority through a gatekeeping role

One of the key concerns expressed by physicians regarding the practice of delayed prescription was the level of autonomy it grants patients, which was perceived as excessively permissive. Participants generally viewed this transfer of decision-making authority as problematic, as it was believed to undermine the professional role and clinical judgement of the physician.

 FG1/P1: I’m afraid that the role of the doctor will increasingly lose its meaning… it will become more and more of a do-it-yourself society. I would really see this as yet another blow. I’d rather make a phone call and get advice after two or three days… Otherwise, it just turns into a prescription grab.

The majority of participating physicians did not believe that the use of delayed prescriptions would significantly impact how patients perceive their professional competence or authority. Most expressed confidence that their clinical judgement would remain respected regardless of the prescription strategy employed. However, a minority of participants expressed concerns that this approach might inadvertently convey uncertainty, leading patients to interpret the delay in prescription as a sign of hesitation or an indication that the doctor is not fully confident in their assessment, potentially undermining the perceived credibility of the medical encounter.

FG5/P3: Yes, I agree, as colleague P4 mentioned, that there could be some ambivalence in how this type of prescription is interpreted. There’s a category of patients who see the doctor as, in a sense, their healthcare leader, right? For them, it might come across as indecision, and therefore not be perceived very positively. And then there’s the more open-minded patient, the one more willing to engage in dialogue, who might see it as a positive thing. So, I think there is a risk, especially with older patients, that the decision to use delayed antibiotic prescription may not be well received.

Participants demonstrated a strong awareness of the legal implications surrounding prescription practices, particularly emphasising that, from a legal standpoint, responsibility for prescribing lies solely with the physician. This awareness underpinned their concerns about granting patients excessive autonomy in the decision-making process. Participants stressed the importance of preserving the professional hierarchy through prescribing practices, asserting that the physician should retain ultimate authority.

FG5/ P4: So, if something happens, as P2 mentioned, the ones that need to be aware of the side effects, it’s us. So, when we prescribe something, we remain responsible. The responsibility is always, and continuously, mine.

Participants demonstrated a clear understanding of the balance between patient autonomy and professional responsibility. None of the physicians opposed shared decision-making and they all acknowledged the importance of aligning the visit with the concerns and preferences of patients. However, they also emphasised that the physician’s role extends beyond merely responding to patient’s preferences; it involves acting as a filter, guiding the patient through available options while maintaining clinical authority. They underscored their role as gatekeepers, managing the integration of patient-acquired knowledge into treatment decisions to ensure both the integrity of medical advice provided and patient safety. Thus, while supporting collaborative communication, they were firm in their belief that the physician should retain the ultimate decision-making power to ensure that the clinical actions remain grounded in medical expertise.

FG2 / P1: It isn’t a bad thing if patients are aware of some medicaments, then it is true that sometimes their sources are not reliable, such as the cousin or the mother-in-law […] I think it works well also if they come spontaneously to request some medicament. Then my role is to talk about it and discuss.

FG4 / P5: Generally I don’t give consent to these requests if I don’t find a clinical indication, but I do so by explaining well to my patients why and how. I always convince them.

### Theme 2: importance of maintaining communication

A key drawback identified by participants regarding the practice of delayed prescription was its potential negative impact on patient–provider communication. The perceived advantage of delayed prescription is that it offers patients greater autonomy, allowing them to self-assess their symptoms and decide whether to fill the prescription, thereby bypassing the need for a follow-up consultation with the doctor. However, our participants highlighted the critical importance of maintaining continuous communication with patients, especially in relation to any changes in treatment plans. They expressed concern that delayed prescriptions might undermine this essential aspect of care by reducing opportunities for dialogue and follow-up, which they view as vital for ensuring optimal patient outcomes.

One of the main objections raised against delayed prescription was its perceived tendency to bypass the doctor–patient interaction. Participants felt that the practice could be seen as a way to avoid engaging in a thorough discussion with the patient, delegating the decision-making regarding treatment to the patient. This, in their view, would diminish the physician’s role in providing expert guidance and fostering a collaborative decision-making process, which they considered central to effective patient care.

FG3 / P3: I think that this delayed prescription is almost like giving a free ticket to our patients, they can do whatever they want […] I prefer as we do now, as the other participant told, after I few days we have to check together the situation.

FG4/ P4: But as my colleagues said, well-informed patients… that’s our job, to educate them and make sure they understand properly. However, I have the impression that with this delayed prescription, even the doctor loses control because they don’t know if the patient actually went to pick up the prescription or not; so, I also struggle to fully understand the concept of this prescription.

FG5 / P4: It has to be very exceptional, it doesn’t mean that the patient has to come back to my office every time, but at least call me to check if the symptoms really call for an antibiotic, for sure I think that we have a responsibility in that sense and we need to maintain it.

Given their heightened awareness of AMR, all participants emphasised the crucial role of effective communication in this context. They stressed that through well-established communication practices, patients can leave consultations with a deeper understanding of their health. When an antibiotic is denied—either during the initial visit or the follow-up consultation—it serves as an educational opportunity. If the patient shows improvement, it provides a chance to reinforce the importance of avoiding unnecessary antibiotic prescriptions. Conversely, if the patient’s condition worsens, the follow-up visit becomes an opportunity to further elaborate on why antibiotics were not deemed necessary initially but became so in the meantime. The visit serves as an ideal platform to enhance the patient’s knowledge about antibiotic prescribing practices, thereby empowering them with greater understanding of the issue.

FG3/ P2: Maybe in the end they don't get the antibiotic, so they shake their heads and go home not very happy, but in my opinion, we must still maintain our position, which is based on scientific evidence, and then, little by little, educate the patients.

FG4 / P1: I think what is more important is to talk and explain to your patient, give them some information.

FG5 / P2: There is always fear, the request comes out of fear when they do not feel well, and that is when the doctor has to step in, to explain and be patient.

Physicians frequently compared the practice of delayed prescribing to the stock antibiotic approach described above. In doing so, they emphasised that the latter places greater importance on communication at the moment of prescription. Since this practice involves an immediately valid prescription, without any enforced delay period, it is the physician’s responsibility, through effective communication and skilled negotiation, to persuade the patient to wait before initiating antibiotic therapy and to begin instead with symptomatic treatment while monitoring the progression of symptoms. Moreover, the stock antibiotic approach entails an additional communicative exchange: physicians typically ask patients to contact them prior to purchasing the medication, should symptoms worsen, reinforcing the collaborative and trust-based nature of this strategy

### Theme 3: healthcare system and guidelines for good practice

One of the key concerns was the perceived inadequacy of delayed prescription within the healthcare system. The widespread use of the stock antibiotic in the region, despite the fact that it is neither formally recognised nor institutionally regulated, led many physicians to view the introduction of delayed prescriptions as redundant. Given the perceived effectiveness and familiarity of the stock antibiotic approach, several practitioners questioned the necessity of implementing an additional prescribing strategy, particularly one that would require changes to established routines and potentially add bureaucratic complexity. Another significant issue that emerged was the waiting period associated with delayed prescriptions. Participants expressed discomfort with the idea of patients potentially experiencing worsening symptoms while left to decide whether to purchase the antibiotic, which they viewed as a problematic and potentially risky aspect of the practice.

While participants acknowledged that, in other healthcare settings, delayed prescriptions might serve as a useful tool to curb antibiotic overprescription by addressing factors such as time constraints, they felt this approach was not directly applicable to their own context. Specifically, they expressed confidence in their ability to schedule follow-up appointments if necessary, preferring this option over leaving the decision entirely in the hands of the patient. In instances where a second appointment was not feasible, participants suggested that they would prefer to keep using the stock antibiotic, without restraining the ability of patients to purchase the antibiotic at any time, and they insisted also on being contacted before the patient purchased the antibiotic, enabling them to reassess the patient’s condition and adjust their diagnosis if needed.

Ultimately, participants perceived delayed prescription as a practice that interferes with their adherence to established professional guidelines and best practices. They emphasised the importance of maintaining control over the decision-making process, viewing the practice as a potential compromise to the quality of care they aim to provide.

FG1 / P1: Also because the wait of three to four days…there are different patients, there is the diabetic one that in one day could get septicemia for an urinary infection and end up intubated.

FG3 / P2: […] I would like to know from the patient why he’s still not feeling well, what has changed and understand if the antibiotic is really the right choice or maybe something else could help. Because to really help our patients we need to know what changed during those days when we have not updated each other.

## Discussion

The results of this study revealed a consistently negative perception of delayed prescription among all participating physicians. The recurrence of certain beliefs and attitudes enabled the identification of several perceived barriers, which were subsequently organised into thematic categories. These barriers, as illustrated in the results and further explored in the subsequent discussion, are multifaceted and span various dimensions of the medical consultation context. The first theme concerns issues of professional authority and the physician’s role in clinical decision-making. The second addresses the relational dynamics between physicians and patients. The third theme pertains to practical and regulatory considerations, including established prescribing routines and physicians’ attitudes toward clinical guidelines promoting responsible antibiotic use. Together, these themes illustrate the complex and interrelated nature of the resistance to delayed prescription, which reflects not only individual-level concerns but also broader systemic and contextual factors.

Before delving into the discussion of the individual themes, there are two broader aspects that were introduced in the results that need to be addressed: the use of the stock antibiotic and the preliminary insights into the broader antibiotic prescription landscape.

### The stock antibiotic

One aspect that emerged as particularly significant is physicians’ perception of the comparison between the stock antibiotic and delayed prescription, which—despite their apparent similarities—are perceived as fundamentally different. Physicians tended to view delayed prescription as a more standardised and routine practice than it actually is. In contrast, when discussing the stock antibiotic, they consistently referred to it as an exceptional measure. It is important to clarify that in countries where delayed prescribing is formally integrated into the healthcare system, it neither replaces immediate prescribing nor eliminates withholding antibiotics altogether. Rather, it serves as an additional option within the physician’s toolkit, allowing clinicians to exercise their professional judgement regarding when and how to use it.^10 15^ A key distinction between the two approaches lies in their flexibility. The stock antibiotic is issued as a regular, immediate prescription, without any predefined waiting period or restrictions that would prevent the patient from obtaining the medication. As a result, it offers greater adaptability to the progression of illness, enabling patients to initiate antibiotic therapy at any point following the consultation.

This comparison, and in particular physicians’ perceptions of the two prescribing practices, is especially significant given the substantial similarities between them. The fact that one approach is perceived far more negatively than the other suggests that even minor procedural or contextual differences can strongly influence physicians’ acceptance and attitudes. These findings underscore the importance of evaluating the implementation of more tailored and context-sensitive approaches when introducing new prescribing strategies.

### Insights on the antibiotic prescription landscape

The current landscape of antibiotic prescription was described by participants as the result of multiple converging factors. Among these, they cited the increased accessibility of health-related information through the internet, which has contributed to greater patient awareness and self-education.^39 40^ Additionally, doctors stated that they perceive the recent experience of the global COVID-19 pandemic as having heightened public interest in understanding of viral infections, thereby influencing attitudes toward the use of antibiotics. Participants also emphasised the impact of regional awareness campaigns conducted in recent years, aimed at educating the public about the risks associated with inappropriate antibiotic use. Finally, the role of ongoing professional education and participation in informative events for healthcare providers was highlighted as a key factor supporting the adoption of more judicious prescribing practices.

The social and cultural context of the region helps explain how participating physicians were able to reflect on and critically compare differing cultural attitudes towards antibiotic use. Their observations were informed not only by their clinical experience but also by their exposure to diverse patient populations, allowing them to identify cultural variations in expectations and behaviours related to antibiotic treatment.

### Theme 1: maintenance of authority through a gatekeeping role

Physicians participating in the study strongly endorsed the authoritative role of the doctor in the clinical decision-making process. This authority was framed not as a means of exerting control or as a matter of status, but as a professional responsibility towards the patient, grounded in clinical expertise and legal accountability. A central concern raised by participants was the shift in decision-making authority inherent in the practice of delayed prescription. In such cases, the responsibility to determine whether or not to initiate antibiotics treatment shifts from the physician to the patient. Participants of our focus groups strongly opposed this transfer of responsibility, arguing that the final decision regarding the provision of medication should remain under the physicians’ jurisdiction.

Participants also emphasised their role as gatekeepers, not only to medication access, but also to the interpretation and application of medical information. While patients receiving a delayed prescription are typically given instructions on how to monitor their symptoms and assess the need for antibiotics, physicians in our focus groups expressed concern about the reliability of such self-assessment and patients’ capacity to interpret their condition accurately. They stressed that the physician’s role involves not only evaluating clinical symptoms but also determining the most appropriate course of action based on medical expertise. Delegating this responsibility to patients was perceived as both superficial and potentially risky, especially considering that the legal liability for the prescription ultimately rests with the physician.

Within this context, the most salient perceived barrier to the implementation of delayed prescription was its potential to undermine professional authority and to dilute the doctors’ responsibility in the prescription process.

### Theme 2: importance of maintaining communication

Participants portrayed the doctor as a professional who must consider the patient’s concerns as a starting point for the visit but should not see their resolution as the only goal; therefore, not allowing those concerns to dictate the course of action. In situations where patients express anxiety or concern, especially when requesting antibiotics in the absence of clear clinical indications, the doctor is expected to serve as a reassuring and informative figure. This involves not only explaining the rationale behind the treatment decision, but also fostering patient understanding to reach a mutually acceptable conclusion. The emphasis was placed on the physician’s ability to guide, educate and collaborate, rather than simply acquiesce to patient demands.

While participants acknowledged the perceived benefits from the patient’s perspective of eliminating the follow-up visit, namely convenience and autonomy, they expressed concerns over the broader clinical and communicative implications of this change. The removal of a scheduled follow-up was seen not merely as the loss of a clinical reassessment opportunity, but as the disruption of an essential communicative process. Check-up appointments were regarded as valuable opportunities in the therapeutic relationship, enabling physicians to engage with patients, assess their progress and, when necessary, refine diagnoses or treatment plans. Thus, while acknowledging the potential benefits of delayed prescriptions in terms of patient autonomy, participants underscored the necessity of maintaining an ongoing, informed relationship with patients to ensure the appropriateness of treatment and to safeguard the quality of care.

The practice of delayed prescription was frequently compared with the one of providing a stock antibiotic. Participants emphasised that the success of the stock antibiotic approach relies heavily on effective communication. Unlike delayed prescription, which includes specific time constraints, the prescription for stock antibiotics is immediately valid and usable; therefore, it falls on the physician to ensure the patient understands the importance of waiting before initiating the treatment. The lack of temporal constraints favour communication also when the patient condition deteriorates, as the patient is expected to call the doctor before using the medication, therefore initiating a further exchange of information and allowing for updated clinical assessment and reinforcing the cautious use of antibiotics.

Importantly, participants emphasised that stock antibiotics are not typically employed as routine substitutes for immediate prescriptions. In contrast, they perceived the delayed antibiotic strategy as a more standardised or habitual alternative. Instead, they are reserved for exceptional situations, such as weekends or instances where a follow-up visit would be logistically challenging due to travel or other constraints. In light of this, a second major limitation of delayed prescription, as perceived by participants, was the potential erosion of meaningful communication and the consequent loss of valuable opportunities for patient education and clinical reassessment.

### Theme 3: healthcare system and guidelines for good practice

One of the main perceived limitations identified by participants concerned the elimination of the follow-up visit, a modification that leads to multifaceted consequences. As previously discussed, the elimination of this second encounter was viewed as detrimental to the communication process, curtailing opportunities for dialogue, reassurance and adjustment of expectations. In the present section, we consider the practical implications of this change, particularly in relation to clinical routines and decision-making processes.

As previously mentioned, the stock antibiotic is considered an exceptional practice, under typical conditions, when there is diagnostic uncertainty, physicians commonly initiate symptomatic treatment and schedule a follow-up consultation within a few days. This second visit serves a critical evaluative function: it allows the clinician to monitor symptom progression and determine whether antibiotic therapy is warranted. If the patient condition has improved, antibiotics are withheld; if symptoms have worsened, further investigations may be pursued, and treatment adjusted accordingly. The follow-up encounter thus constitutes an integral component of what many participants considered good clinical practice, enabling a more cautious, evidence-based approach to prescribing.

Participants strongly valued this follow-up as a safeguard for good clinical practice, emphasising its importance in enabling cautious and responsive prescribing. The removal of this step, as often implied in delayed prescription strategies, was therefore viewed as a significant practical limitation. It was seen as reducing the physician’s ability to monitor the evolution of the illness and intervene appropriately.

This resistance was particularly evident when participants compared delayed prescription with the alternative strategy of the stock antibiotic, although both strategies aim to reduce unnecessary antibiotic use by postponing the initiation of treatment, participants highlighted key differences in how control over clinical decision-making is distributed between doctor and patient.

In the case of the stock antibiotic, patients are typically informed that the prescription is not to be used autonomously but remains contingent on further instruction from the physician, generally following a call to the doctor’s office. This approach allows doctors to preserve a high degree of control over the course of treatment and to monitor the progression of symptoms before authorising antimicrobial therapy. Patients, in this dynamic, assume the role of collaborators who contribute feedback and symptom updates, enabling an ongoing, interactive process of clinical evaluation.

The preference for the stock antibiotic strategy was rooted in pragmatic considerations. Participants described it as more efficient, better aligned with their usual practices and more consistent with a cautious approach to prescribing. This pre-existing preference contributed to a general lack of enthusiasm for adopting delayed prescription more broadly, despite its potential benefits.

## Conclusions

Even though our physicians recognised the benefits of delayed prescription of antibiotics, they also described it as a practice that is functional depending on the surrounding and different social factors, such as the workload family doctors experience, the influence of patient’s demand and time constraints. In their view, delayed prescription could not be indiscriminately implemented, and they highlighted a few domains that would be damaged in their regional context if that was the case. In particular, what resulted is that their current practice aligns more with the idea they share of antibiotic use and diagnostic meticulousness, as they believe it allows more dialogue and social interaction.

Findings from the focus groups indicate that delayed prescription is perceived to be a threat to doctors’ authority. Patients’ independent evaluation is not highly valued and discarded in favour of major control from the doctor’s side, recalling a dynamic of paternalism.

Even considering that delayed prescription is to be used only in case of very mild infection, and it does not substitute immediate prescription, GPs demonstrated great apprehension for the conditions of their patients. Today’s interactions in the medical context are based on the concept of informed consent and patient’s autonomy,^41^ but the reactions gathered through our focus groups recall a more paternalistic approach.

The opinions that emerged in this study diverge completely from the line of results reported by ongoing research on delayed prescription and its benefits, as reported in the introduction. In similar studies performed in Malta^42^ and Norway,^43^ GPs were able to identify some benefits of the practice, especially for what concerns the chance of educating patients on cautious antibiotic usage and the empowering effect it provides on patients. The results of our study demonstrate that doctors from the Italian-speaking region of Switzerland do not recognise these positive aspects, nor do they seem interested in implementing them in their practice.

Results from this study align with those of a recent review,^12^ which identified nine key context–mechanism–outcome configurations (CMOCs) that help explain physicians’ decision-making processes regarding antibiotic prescribing. Notably, the attitudes expressed by the family doctors in our study correspond to several of the contextual factors outlined in that review. The authors of the review grouped these CMOCs into four overarching themes: the value of delayed prescription as a safety net, patient concordance and GP self-efficacy, the value of delayed prescription as a social tool, and the influence of the work environment. Of these, the first three were also reflected in themes that emerged from our analysis. Specifically, many of the reasons cited by participants for their reluctance to adopt delayed prescription mirrored barriers outlined in the review. These included: the belief that requiring a follow-up visit is a safer approach; uncertainty about whether patients will adhere to instructions; perceptions that existing guidelines do not support delayed prescribing; concerns that delayed prescription might convey mixed messages about antibiotic use; preference for using communication skills to justify non-prescription decisions directly; fear that the strategy might undermine professional credibility or patient trust; and apprehension that delayed prescriptions could reinforce patients’ misconceptions about the appropriateness of antibiotics for self-limiting illnesses.^12^

### Practical implications

Results from this study highlight the necessity of further investigating and understanding GPs’ attitudes towards prescribing practices. The findings also demonstrate the key role of doctors in successfully introducing a new practice: regardless of its potential benefits, if GPs are not supportive, they may simply disregard it, thereby hindering any possibility of experiencing the benefits.

A relevant implication emerging from the recurring comparison between stock antibiotics and delayed prescriptions concerns the existence and use of an informal, unregulated practice in the region. Through physicians’ accounts, we became aware of the widespread use of the stock antibiotic strategy, typically adopted in clinical situations where delayed prescriptions are employed in other countries. While both approaches share many similarities, the main distinguishing feature lies in the greater flexibility of the stock antibiotic: it does not require patients to adhere to a predefined waiting period, allowing for a more personalised adaptation to the individual course of illness.

The fact that physicians perceive the two practices so differently, despite their structural resemblance, underscores the importance of conducting qualitative research with target populations to identify their specific needs and preferences. In this context, a key practical implication may not be the introduction of delayed prescription as a formal practice—given its negative perception among physicians—but rather the potential regulation and formalisation of the stock antibiotic strategy, which appears to function as a more tailored version of delayed prescribing and aligns more closely with the attitudes and clinical routines of local healthcare professionals.

If delayed prescription were to be introduced in Switzerland or in a similar country where it has not yet been implemented, the findings of this study highlight the importance of involving family physicians in the development of tailored legislation that aligns the practice with local needs. In particular, it would be beneficial to establish a system in which the patient is required to communicate—at least via phone—with the physician before the prescription becomes valid and eligible for dispensing.

Furthermore, family physicians could benefit from targeted informational sessions on the effectiveness of delayed prescription, particularly focusing on evidence regarding patient satisfaction and the lack of significant adverse health outcomes associated with its use. Emphasising these aspects may help address some of the concerns expressed by physicians, thereby fostering a more favourable attitude towards the implementation of the practice.

This study is part of a larger project, future studies aim to quantitatively explore the relationship between patient and doctor’s trust and the influence it may have on delayed prescription acceptance. The focus on delayed prescription is central to the project due to the effectiveness of the strategy in curtailing antibiotic use while efficiently treating respiratory tract and urinary tract infections.

### Strengths and limitations of the study

The attitudes of our participants could be shaped by the context in which they operate: as they also recognised, the Italian-speaking part of Switzerland is a small region and, depending on the office location, there could be social dynamics more similar to those of small towns than big cities. The closeness and bond shared between family doctors and their community is one regional element to take into consideration while evaluating the attitudes of our sample. As the study explored delayed prescription in a context where the practice is not currently in use, participants’ insights were based on perceptions and expectations rather than direct experience.

One strength of the study is the absolute concordance of opinion expressed by the participants: even though they operate in different contexts, ranging from valleys to cities, they all shared the same stance without hesitation.

One limitation could lie in the modality of the focus groups as doctors might display less of a liberal approach when confronted with peers and show a more diligent adherence to guidelines. Another limitation can be found in the selection of doctors, since the participation in focus groups was on a voluntary basis, it is possible that those that accepted are the ones that were already sensitive to the theme.

## Supplementary material

10.1136/bmjopen-2024-094296 online supplemental file 1

## Data Availability

All data relevant to the study are included in the article or uploaded as supplementary information.
