# Supplementary material for: Qualitative study on doctors’ perceived barriers and facilitators towards the practice of delayed prescription: results from focus group discussions in Switzerland
Source: BMJ Open. 2025 Nov 4;15(11):e094296. doi: 10.1136/bmjopen-2024-094296 (PMC12587955; doi:10.1136/bmjopen-2024-094296)
Supplement: online supplemental file 1 [file bmjopen-15-11-s001.docx]

**SUPPLEMENTARY MATERIAL A:**

Guided track for focus groups:

Introduction

*Hello everyone,*

*My name is … and I will be your moderator today. Here with me there is an assistant, her name is …*

*My role as a moderator is to guide the discussion.*

*Today discussion will touch upon your thoughts on antibiotics and a practice called delayed prescription, we will talk about that later. Our objective is to uncover your opinions in an open discussion where anyone should feel free to share their thoughts.*

*The results gathered here today will inform a questionnaire that will be distributed among patients.*

*Any of the information discussed today is gathered with the intention of modifying practices that are in use at the moment.*

*This study is part of a bigger project funded by the Swiss National Science Foundation. The project focuses on the element of trust in the patient-provider relationship in the specific context of antibiotic prescription.*

*We are not in contact with pharmaceuticals and any entity of public health services, the aim of our research does not include informing them of the results.*

*We already performed some focus groups with the population on the same topic and we are now interested in gathering the opinions of health experts.*

The “Rules”

*There are no right or wrong opinions on the topic, there are only different point of view. We are looking forward to hearing your opinions and we hope it will be an interesting discussion. There might be some cases where you do not agree with the others, but it is important to still listen to build a constructive discussion. If something is not clear, you can ask question at any time.*

*Today’s discussion will be recorded, only the researchers working on the project will have access to the recordings. Recordings will be deleted after the transfer on a computer, and data on the personal information of participant will be censored. A code will be applied to each participant to anonymize their identity.*

Introduction of participants (1 min each)

*To get to know us better, we will go in turns and each one of us will tell some basic knowledge on themselves.*

Theme 1: Antibiotics

*As mentioned before, today’s exchange will be on the topic of antibiotic prescription and delayed prescription. We want to gather your opinions on those topics:*

*- What is your typical experience of prescribing antibiotics to your patients?*

*- What is the general attitude of your patients towards antibiotic use?*

*- Are there any differences in your patient’s attitudes that are due to gender, age or any other characteristics?*

*- How would you describe the efficacy of antibiotics? What kind of side effects have been reported to you by your patients?*

*- What are your thoughts on your patients’ adherence to the antibiotic treatment?*

*- Do you reckon it is possible to understand if a patient did not adhere to the treatment? What are the signs? And how would you react in this situation?*

*- What would you describe as the reason for a patient to not adhere to an antibiotic therapy?*

*- Has it ever occurred that a patient would insistently ask for an antibiotic? How would you manage this situation? How do you behave when you believe that the antibiotic is not the right solution?*

*- Has it ever occurred to you that a patient comes to the office for an antibiotic prescription after having purchased the medicament on the pharmacist’s indications?*

*- How would you rate your patients’ knowledge on the topic of antibiotics?*

*- Do you think your patients know about antimicrobial resistance and its development?*

*- How can we oppose to antimicrobial resistance?*

Theme 2: Delayed prescription of antibiotics

*Up until now, we have discussed on antibiotics. Now we would like to talk about delayed prescription.*

*- Has any of you already heard of delayed prescription of antibiotics? If not, what kind of practice would you imagine it to be?*

*We will now give an explanation to make sure that we are all thinking about the same practice:*

*Delayed prescription is a prescription that is issued by the doctor after the visit, in those cases where an infection is spotted. The delayed prescription can be collected and used by the patient only after a few days (usually from three to five). Usually, the doctor would leave the prescription at the secretary’s desk and the patient can come back to collect it, without going through a second visit with the doctor, in case the symptoms have not gotten better or have not improved.*

*This practice is not allowed in Switzerland, but we want to hear about your opinions on it.*

*- What are your thoughts on this practice? If it was available in Switzerland, would you use it with your patients?*

*- Are there any categories of patients that would be more suitable for receiving this type of prescription? Conversely, which kind of patient would be less adequate?*

*- What are the specific characteristics influencing the suitability of a patient to receive a delayed prescription?*

*- What are the specific contexts, or symptoms for which you would use this prescription? What would be the contexts or symptoms for which you would not use this prescription?*

*- In your opinion, for what types of symptoms would your patients be more likely to accept a delayed prescription? And for which symptoms would they be less likely?*

*- In your opinion, what would your patients think about you if you would use this type of prescription and why? Do you think your patients would be satisfied?*

*- How would you expect the symptoms to evolve during the waiting days? Would expect and improvement, a worsening, or a stable situation?*

*- Would your prognosis be affected by the availability of a practice such as delayed prescription?*

Final evaluation on delayed prescription

*- I would like to hear from you what you think would be the main benefits coming from a practice like delayed prescription. What could it be useful for? What benefits could it bring to patients? To doctors? To society? What kind of positive effects could it have? Why?*

*- Conversely, I would like to discuss about the downsides. What kind of negative effects could it have on the patients? On the doctors? On society? Why?*

Theme 3: Patients’ requests

*Before ending the discussion, we would like to add a few questions on you interaction with patients, especially on the topic of requests:*

*- Do usually patients come to your office requesting medicaments? How often would that happen and what kind of medicaments would they typically request?*

*- Do you see the request as something that facilitates the interaction or as something that can make the visit more complicated?*

*- Do you believe there should be some class of medicaments that patients should not be allowed to request from you?*

*We have reached the end of our discussion.*

*I would like to thank you all for participating, you input is extremely valuable for our research.*

*You will receive a report on the results of the focus groups.*

*Thank you again.*
