# Supplementary material for: Qualitative study on doctors’ perceived barriers and facilitators towards the practice of delayed prescription: results from focus group discussions in Switzerland
Source: BMJ Open. 2025 Nov 4;15(11):e094296. doi: 10.1136/bmjopen-2024-094296 (PMC12587955; doi:10.1136/bmjopen-2024-094296)
Supplement: Supplementary file 2 [file bmjopen-2024-094296.reviewer_comments.pdf]

PEER REVIEW HISTORY

BMJ Open publishes all reviews undertaken for accepted manuscripts. Reviewers are asked to complete a checklist review form and are provided with free text boxes to elaborate on their assessment. These free text comments are reproduced below.

ARTICLE DETAILS

Title (Provisional)

A qualitative study on doctors' perceived barriers and facilitators towards the practice of delayed prescription. Results from focus group discussions

Authors

Rinaldi, Aline; Petrocchi, Serena; Bullo, Anna; Gabutti, Luca; Schulz, Peter

---

VERSION 1 - REVIEW

**Reviewer**	**1**
**Name**	**Bakhit, Mina**
**Affiliation**	**Bond University, Centre for research in Evidence-Based Practice**
**Date**	**27-Feb-2025**
**COI**	**None**

Thank you for the opportunity to review this manuscript. This is a well-written qualitative study to explore the main barriers and facilitators by GPs to use delayed prescribing.

Major comments:

1. Methods: If delayed prescribing is not allowed, then how the GPs would be able to know the barriers or facilitators of its implementation? Maybe it is more about perceived barriers and facilitators, including healthcare system-related barriers.

2. Results: Please write the means and standard deviation in full, when first mentioned

3. Results: A major concern that I have is that the results are not clearly divided into themes (and subthemes). I expected to see this as the authors mentioned that they had conducted a thematic analysis. I recommend restructuring the Results to themes/subthemes with relevant quotes clearly reflecting the themes and editing the Results section of the Abstract accordingly.

4. Discussion: update the summary of findings after clarifying the themes/subthemes

5. Discussion, limitations: Highlight that the no use of delayed prescribing could be a limitation for GPs to provide experience-based feedback.

Minor Comments

6. Introduction: It is a bit unclear why delayed prescribing is not allowed. Any reference to support this would be helpful.

7. Introduction: minor referencing typos, please review the Introduction and throughout.

8. Introduction, lines 29-31: the sentence contradicts the previous paragraph about the impact and future death toll. I recommend adding this sentence before the statement about AMR death and highlighting the impact of antibiotic resistance on healthcare-associated costs, etc

9. Introduction, line 40: Please edit to present what type of patients could benefit from delayed prescribing. As we do not have sufficient data to cover “most patients” and conditions.

10. Introduction, lines 8-9: add supporting references

11. Introduction and in other sections: please be consistent when referring to either GPs or healthcare professionals

12. Methods: Please clarify if the GPs have received any kind of incentives for their participation or not.

**Reviewer**	**2**
**Name**	**Khouja, Tumader**
**Affiliation**	**University of Florida**
**Date**	**04-Apr-2025**
**COI**	**None**

This qualitative study addresses physician’ s barriers to adopting delayed antibiotic prescribing, an antimicrobial stewardship strategy, in a region in Switzerland. The study is well written and displays the concerns physicians have in adopting this strategy.

Comments to strengthen the paper:

-In the background, it would be helpful to add in information regarding the magnitude of AMR in Switzerland. Does the country have an AMR plan? What are the components of the program.

-The participants mention cultural differences in patient demand for antibiotics. It would be helpful for international readers to have a brief description of the cultural make-up of the region investigated.

- There are multiple quotes from the same participants through the paper (FG5/P4), which makes it sound like this participant was dominating the conversation. I would recommend diversifying the respondent quotes.

-when reporting quotes, it is recommended to add in the demographics of the participant. For example, after a quote add in parentheses participants (sex, age, years in practice). This also helps identify patterns by demographics if there are any.

-The authors state that the practice of delayed prescribing is not legal in Switzerland, which would be the biggest barrier to implement this practice. As mentioned in the paper, many of the physicians reported recent education, workshops and following guidelines when it comes to prescribing antibiotics. What interventions would the authors propose to help physicians understand and implement this practice? What advantage would delayed prescribing have over the current practice of stock antibiotics? Is it patient autonomy only?

-Can the authors add a brief description on how prescriptions are written in this region? Are prescriptions in this region made electronically? Would it be possible for a physician to place an electronic order for a delayed prescription and receive a notification when a patient picks it up?

- I would recommend the authors review this article as it relates to the discussion

Mcleod, M., Campbell, A., Hayhoe, B. et al. How, why and when are delayed (back-up) antibiotic prescriptions used in primary care? A realist review integrating concepts of uncertainty in healthcare. BMC Public Health 24, 2820 (2024). https://doi.org/10.1186/s12889-024-20248-8

VERSION 1 - AUTHOR RESPONSE

**Reviewer 1:**<br>Dr. Mina Bakhit, Bond University	
1. Methods: If delayed prescribing is not allowed, then how the GPs would be able to know the barriers or facilitators of its implementation? Maybe it is more about perceived barriers and facilitators, including healthcare system-related barriers.	We have better specified that the focus of our research are the perceived barriers and limitation that doctors would face in case delayed prescription was to be implemented in a context where there is no prior use of the practice.
2. Results: Please write the means and standard deviation in full, when first mentioned.	The means and standard deviation have been written in full when first mentioned

3. Results: A major concern that I have is that the results are not clearly divided into themes (and subthemes). I expected to see this as the authors mentioned that they had conducted a thematic analysis. I recommend restructuring the Results to themes/subthemes with relevant quotes clearly reflecting the themes and editing the Results section of the Abstract accordingly.	The result section has been updated to show three main themes, each completed by the quotes gathered during the focus groups.
4. Discussion: update the summary of findings after clarifying the themes/subthemes.	The discussion has been updated to present a first part with a comment for each theme highlighted in the results, and in the second part a more general comment.
5. Discussion, limitations: Highlight that the no use of delayed prescribing could be a limitation for GPs to provide experience-based feedback.	The objective of the study was to explore the perceived barriers and facilitators to adopting delayed prescribing in a context in which DP is not yet allowed. This focus was clarified and consistently reflected in the manuscript, where the term perceived was used to specify the nature of the insights gathered. In this sense, the lack of direct experience does not weaken the findings, but rather aligns with the study's aim of anticipating potential challenges and opportunities prior to wider adoption of the practice.
6. Introduction: It is a bit unclear why delayed prescribing is not allowed. Any reference to support this would be helpful.	There is no formal law prohibiting to use delayed prescription, but the tools doctors have to prescribe medication, do not include a prescription that becomes available at a later date. We have rephrased some section to make the situation clearer.

7. Introduction: minor referencing typos, please review the Introduction and throughout.	The whole paper has been checked and corrected.
8. Introduction, lines 29-31: the sentence contradicts the previous paragraph about the impact and future death toll. I recommend adding this sentence before the statement about AMR death and highlighting the impact of antibiotic resistance on healthcare-associated costs, etc.	The paragraph has been formulated again to give a more logic progression of the burden caused by AMR.
9. Introduction, line 40: Please edit to present what type of patients could benefit from delayed prescribing. As we do not have sufficient data to cover "most patients" and conditions.	The paragraph was edited to better specify the type of patients that benefited from delayed prescription.
10. Introduction, lines 8-9: add supporting references.	The whole text has been checked and some more supporting references have been added.
11. Introduction and in other sections: please be consistent when referring to either GPs or healthcare professionals.	To have more clarity on the professional figure mentioned we used a selection of synonyms, such as GP, family doctor, physicians, practitioner, etc. and we have modified any use of "healthcare professional" as it is too vague.
12. Methods: Please clarify if the GPs have received any kind of incentives for their participation or not.	We have added information on the doctor's incentives that were previously established by the ethical committee.

Reviewer 2:

Dr. Tumader Khouja, University of Florida

1. In the background, it would be helpful to add in information regarding the magnitude of AMR in Switzerland. Does the country have an AMR plan? What are the components of the program.

In the background we have added some information on the current local situation on AMR.

2. The participants mention cultural differences in patient demand for antibiotics. It would be helpful for international readers to have a brief description of the cultural make-up of the region investigated.

The background has been modified to include some brief information on the local context and. A brief overview of the population has been added in the discussions.

3. There are multiple quotes from the same participants through the paper (FG5/P4), which makes it sound like this participant was dominating the conversation. I would recommend diversifying the respondent quotes.

The quotes have been diversified more and now direct quotes of FG5/P4 appear less often.

4. when reporting quotes, it is recommended to add in the demographics of the participant. For example, after a quote add in parentheses participants (sex, age, years in practice). This also helps identify patterns by demographics if there are any.

We agree that including demographic information alongside participant quotes can sometimes enrich qualitative interpretation. However, in our case, we chose not to do so because the participants consistently expressed very similar views and shared attitudes, forming a unified front in their perceptions of delayed prescription. As such, no meaningful patterns or differences emerged that could be linked to participant age or other demographic characteristics. We therefore felt that attaching this information would not add interpretive value to the findings and might even suggest distinctions that were not present in the data.

5. The authors state that the practice of delayed prescribing is not legal in Switzerland, which would be the biggest barrier to implement this practice. As mentioned in the paper, many of the physicians reported recent education, workshops and following guidelines when it comes to prescribing antibiotics. What interventions would the authors propose to help physicians understand and implement this practice? What advantage would delayed prescribing have over the current practice of stock antibiotics? Is it patient autonomy only?

We have now better clarified that it is not illegal to use delayed prescription, but there is no practical tool to delay the availability of a prescription at the moment. The prior wording was more rigid, and we believe that it resulted in an exaggerated depiction of the situation. We have now rephrased the relevant sections to make the context clearer.
In the discussion we added a small section on the practical implication of the results of this study where we answered the questions here reported

6. Can the authors add a brief description on how prescriptions are written in this region? Are prescriptions in this region made electronically? Would it be possible for a physician to place an electronic order for a delayed prescription and receive a notification when a patient picks it up?

In the introduction a brief explanation of the prescribing practices has been added, and throughout the paper we better specified the modalities employed by doctors for antibiotic prescription.

7. I would recommend the authors review this article as it relates to the discussion Mcleod, M., Campbell, A., Hayhoe, B. et al. How, why and when are delayed (back-up) antibiotic prescriptions used in primary care? A realist review integrating concepts of uncertainty in healthcare.
BMC Public Health 24, 2820
(2024). https://doi.org/10.1186/s12889-024-20248-8

The paper was read by the authors and has been referenced in our study adding a comparison of the results in the discussion.
